# Current Understanding of Bovine Ketosis: From Molecular Basis to Farm-Level Management

**DOI:** 10.3390/ani15243644

**Published:** 2025-12-18

**Authors:** Yigang Zhang, Changfa Wang, Muhammad Zahoor Khan, Zhihua Ju, Jinming Huang

**Affiliations:** 1 Liaocheng Research Institute of Donkey High-Efficiency Breeding and Ecological Feeding, College of Agriculture and Biology, Liaocheng University, Liaocheng 252000, China; 2 Key Laboratory of Livestock and Poultry Multi-Omics of MARA, Institute of Animal Science and Veterinary Medicine, Shandong Academy of Agricultural Sciences, Jinan 250100, China

**Keywords:** cow, negative energy balance, ketosis, candidate genes, therapeutic

## Abstract

Bovine ketosis represents a critical metabolic challenge in modern dairy production, primarily affecting high-yielding cows during the periparturient period. The disorder arises from severe negative energy balance (NEB), where energy demands exceed supply, triggering substantial mobilization of adipose tissue and hepatic accumulation of non-esterified fatty acids (NEFAs). This stress overwhelms hepatic oxidative capacity, leading to incomplete fatty acid oxidation and excessive ketone body production—primarily β-hydroxybutyrate (BHB), acetoacetate, and acetone. While genomics enables the identification of candidate genes and genetic variations influencing ketosis susceptibility, metabolomics provides comprehensive, multi-matrix metabolic signatures across blood, milk, urine, and tissues, often revealing predictive biomarkers in the dry period and pre-clinical stage. This review provides a comprehensive overview of ketosis etiology, emphasizing the roles of NEB, fat mobilization, hormonal dysregulation, and oxidative stress. We systematically examine candidate genes, signaling pathways, and the diverse metabolomic profiles underlying disease pathogenesis, and discuss current prevention strategies focusing on periparturient nutritional management and therapeutic interventions. Understanding these integrated molecular and metabolic mechanisms offers valuable insights for developing targeted breeding programs and precision management approaches to enhance metabolic health and economic efficiency in dairy cattle.

## Simple Summary

This comprehensive review examines bovine ketosis from molecular mechanisms to practical farm management. The article explores how high-producing dairy cows experience ketosis when energy demands exceed intake during early lactation, leading to fat mobilization and excessive ketone production. Through detailed analysis of genetic determinants, including genes controlling lipid metabolism, ketone synthesis enzymes, and transcriptional regulatory factors, the review bridges fundamental metabolic pathways with applied strategies. Current prevention approaches emphasizing periparturient nutrition and therapeutic interventions are evaluated, providing valuable guidance for improving dairy cow health and production efficiency.

## 1. Introduction

**Figure 1:**
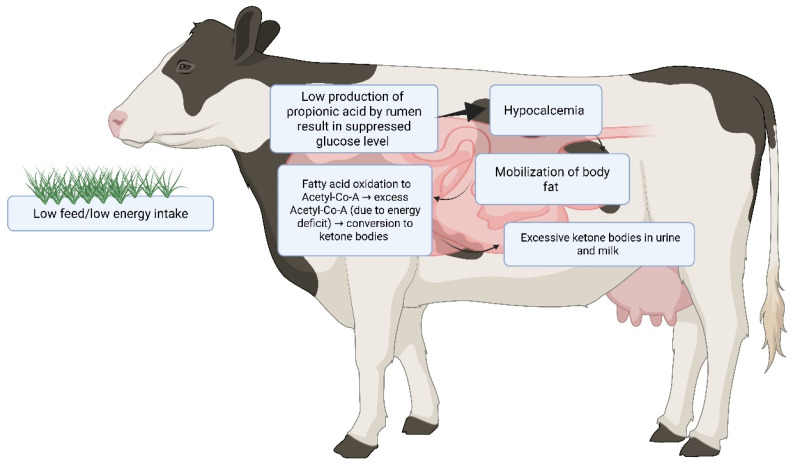
Schematic illustration of the pathophysiological cascade leading to bovine ketosis in dairy cattle. The figure illustrates that low feed intake triggers hypoglycemia and body fat mobilization. Hepatic fatty acid oxidation produces excess acetyl-CoA, which is converted to ketone bodies (pink area shows affected organs). Ketone overproduction results in hyperketonemia with excretion in urine and milk, often accompanied by hypocalcemia.

The periparturient period in dairy cattle, spanning approximately three weeks before to three weeks after calving, represents a critical transition phase characterized by dramatic metabolic and physiological changes [1,2]. During this time, cows face the challenge of rapidly increasing milk production while experiencing decreased dry matter intake [3,4]. This metabolic imbalance creates a negative energy balance (NEB), where energy demands for milk synthesis exceed dietary energy supply (Figure 1) [4,5]. To offset this deficit, cows mobilize body fat reserves, releasing non-esterified fatty acids (NEFAs) into circulation. The liver uptakes these NEFAs, but when the influx overwhelms its oxidative capacity, incomplete oxidation occurs, producing ketone bodies—primarily beta-hydroxybutyrate (BHB), acetoacetate, and acetone [2]. Ketosis develops when ketone body production exceeds utilization, typically occurring 2–6 weeks postpartum when milk production peaks. Although NEB is the major metabolic driving factor, evidence also indicates that hyperketonemia is not merely a consequence of energy insufficiency. Systemic inflammation, oxidative stress, and the accumulation of toxic lipid intermediates may act as additional contributing factors that further disrupt hepatic and peripheral insulin signaling, thereby aggravating the development of ketosis.

Intensive genetic selection and improved feeding systems have markedly increased milk yield in modern dairy cows, particularly through the selection of high-producing genotypes, enhancement of feed intake capacity, and optimization of nutrient utilization efficiency [6,7,8]. Genetic selection programs, driven by systematic recording and genomic evaluation, have continuously prioritized traits associated with lactation performance, leading to substantial gains in average milk yield across generations [9,10]. At the same time, advances in feeding systems—especially precision-formulated total mixed rations (TMR), higher dietary energy densities, and improved forage quality—have further supported the elevated metabolic requirements for sustained high milk output [11]. As a consequence, modern dairy cows now often produce 8000–12,000 L per year (compared to about 2000–3000 L per year in the 1950s), but this marked increase in milk yield is accompanied by a considerable rise in metabolic load [8,12]. During early lactation, the sharp rise in energy demand frequently induces NEB, a condition in which the energy demand of the mammary gland exceeds dietary intake [13,14], predisposing cows to metabolic disorders [15,16]. Among these, ketosis represents the most prevalent and severe condition, posing a major threat to the health and productivity of high-yielding dairy herds.

Clinical ketosis signs include reduced feed intake, weight loss, decreased milk yield, and a characteristic sweet breath odor. Subclinical ketosis, affecting up to 40% of dairy cows, often goes undetected but significantly impacts productivity and predisposes cattle to other metabolic disorders, which can progress to secondary complications such as mastitis, metritis, and hypocalcemia [17,18]. Based on blood β-hydroxybutyrate (BHB) concentrations and clinical manifestations, ketosis is categorized as either subclinical (SCK) or clinical (CK): SCK is defined by BHB ≥ 1.2 mmol/L without visible symptoms, whereas CK involves BHB ≥ 3 mmol/L accompanied by clinical signs [19,20,21]. However, reported thresholds vary between studies (blood BHB thresholds for SCK range approximately 1.0–1.4 mmol/L), and some recent investigations and monitoring protocols advocate for lower warning thresholds (BHB ≥ 1.0 mmol/L) or classify values ≥ 1.5 mmol/L as high-risk in high-yielding Holstein–Friesian cows. The variation in threshold selection reflects differences in breed, production level, days in milk (DIM), and diagnostic methods [11,22]. Crucially, the correlation between BHB concentration and clinical severity is not absolute; some cows with extremely high BHB levels remain clinically normal, while others with mild hyperketonemia are severely sick [23]. This clinical heterogeneity implies that ketone bodies are likely not the sole pathological agents. Instead, the full manifestation of the disease may involve the perturbation of other metabolites, microRNA, or the systemic effects of inflammation, suggesting that the precise molecular trigger for clinical ketosis is still an area of intensive investigation.

Clinically and metabolically, ketosis does not represent a single homogeneous disorder; rather, at least two distinct forms are recognized in dairy cows. Type I ketosis (nutritional or “classic” ketosis) typically develops 2–6 weeks postpartum, when peak milk yield creates a sharp energetic deficit. It is characterized by markedly elevated BHB and NEFA concentrations, accompanied by hypoglycemia due to insufficient hepatic gluconeogenesis [24]. In contrast, Type II ketosis (fatty liver–associated ketosis) usually occurs within the first 1–2 weeks postpartum and is linked to excessive hepatic fat accumulation and impaired liver function [25]. Type II cows often exhibit elevated BHB and NEFA without severe hypoglycemia, and may present hyperinsulinemia, impaired insulin signaling, and even hyperglycemia, reflecting systemic insulin resistance [25]. Thus, although both types share hyperketonemia as a metabolic feature, their underlying mechanisms and metabolic signatures differ substantially; Type I is primarily driven by negative energy balance, whereas Type II involves hepatic dysfunction and altered endocrine regulation [24]. Because these pathophysiological routes are distinct, they likely involve partly divergent molecular networks, which must be considered when interpreting biomarker profiles, metabolic pathways, and genetic regulation in the context of ketosis.

In intensive dairy production systems, ketosis has a high incidence rate and causes considerable annual economic losses [26]. Current prevention strategies mainly focus on periparturient feeding management, maintaining appropriate body condition, and implementing early monitoring programs to minimize the severity of NEB and subsequent ketone body accumulation [27,28,29,30]. Nonetheless, variations in management and production systems mean that ketosis remains inadequately controlled in many regions worldwide [31].

Genetic variations with additive effects exist in the indicator metabolites of ketosis, which can be exploited through genetic selection programs to reduce the incidence of ketosis in dairy cows [32]. Therefore, elucidating the molecular mechanisms and regulatory networks underlying ketosis is of great importance for improving metabolic health and production efficiency in dairy cows. Recent advances in molecular biology have enabled the identification of key metabolic pathways and regulatory genes involved in the disease, including those related to fatty acid oxidation, gluconeogenesis, insulin signaling, and hepatic energy metabolism. These insights offer new opportunities for understanding the pathogenesis of ketosis and for developing targeted prevention strategies.

## 2. Etiology and Pathophysiological Mechanisms of Bovine Ketosis

The development of ketosis in dairy cows represents a complex cascade of metabolic disturbances initiated by energy imbalance and perpetuated through interconnected pathways involving lipid mobilization, hepatic dysfunction, hormonal alterations, and oxidative stress. Understanding these multifaceted mechanisms is essential for developing effective prevention and intervention strategies.

### 2.1. Negative Energy Balance and Adipose Tissue Mobilization

During early lactation, the metabolic demands for milk synthesis increase dramatically, often exceeding the cow’s capacity to consume sufficient energy through feed intake [31,33,34,35]. This physiological mismatch creates a state of NEB, wherein energy expenditure surpasses dietary energy supply. In response to this energetic deficit, the cow initiates a compensatory mechanism by mobilizing endogenous energy reserves stored in adipose tissue [32]. Specifically, hormone-sensitive lipase and adipose triglyceride lipase catalyze the hydrolysis of triglycerides, liberating substantial quantities of NEFAs into the systemic circulation [32]. These NEFAs are subsequently transported to the liver, where they serve as primary substrates for energy production through β-oxidation [36].

However, the bovine liver possesses inherent metabolic limitations that predispose high-yielding cows to ketosis. Unlike monogastric species, ruminant hepatocytes exhibit markedly reduced capacity for complete fatty acid oxidation and possess extremely limited ability to export triglycerides via very-low-density lipoprotein (VLDL) particles [37]. Consequently, when NEFA influx overwhelms hepatic oxidative capacity, incomplete β-oxidation occurs, resulting in two detrimental outcomes: intracellular lipid accumulation manifesting as fatty liver disease, and excessive production of ketone bodies [37]. This dual pathology establishes the metabolic foundation for both hepatic dysfunction and systemic ketosis.

### 2.2. Hepatic Ketogenesis and Ketone Body Accumulation

**Figure 2:**
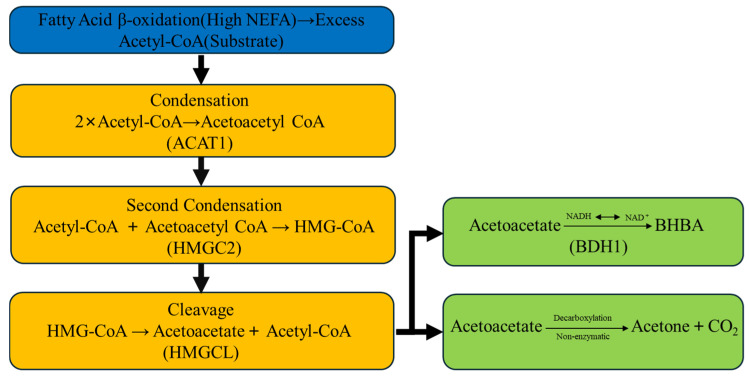
Schematic representation of the hepatic ketone body synthesis pathway in dairy cows during negative energy balance.

The excessive hepatic uptake of NEFAs triggers upregulation of key ketogenic enzymes, fundamentally altering hepatic metabolism toward ketone body synthesis. Central to this process are two rate-limiting enzymes: 3-hydroxy-3-methylglutaryl-CoA synthase 2 (HMGCS2) and HMG-CoA lyase (HMGCL), which catalyze critical steps in the ketogenic pathway [38]. As illustrated in Figure 2, the ketogenic cascade begins when acetyl-CoA molecules derived from incomplete fatty acid oxidation undergo enzymatic condensation by acetyl-CoA acetyltransferase 1 (ACAT1) to form acetoacetyl-CoA. Subsequently, HMGCS2 catalyzes the condensation of acetoacetyl-CoA with another acetyl-CoA molecule to generate 3-hydroxy-3-methylglutaryl-CoA (HMG-CoA) [39]. HMGCL then cleaves HMG-CoA to produce acetoacetate and acetyl-CoA [40]. Finally, β-hydroxybutyrate dehydrogenase 1 (BDH1) reversibly converts acetoacetate to β-hydroxybutyrate (BHB), while acetoacetate can also spontaneously decarboxylate to form acetone [41,42,43].

These ketone bodies, including BHB, acetoacetate, and acetone, normally serve as crucial alternative energy substrates for peripheral tissues, particularly during periods of glucose scarcity. Extra-hepatic tissues such as skeletal muscle, cardiac muscle, and brain utilize ketone bodies through oxidative metabolism, thereby sparing glucose for essential metabolic processes [44]. However, when the rate of hepatic ketone production substantially exceeds the capacity of peripheral tissues for ketone utilization and renal excretion, systemic accumulation occurs, leading to hyperketonemia—the biochemical hallmark of clinical ketosis [45]. In severe cases, this metabolic derangement progresses to ketoacidosis, characterized by metabolic acidosis and profound systemic consequences (Figure 1).

### 2.3. Hormonal Dysregulation and Metabolic Signaling Disruption

The periparturient period is characterized by profound alterations in the endocrine milieu, which play a pivotal role in orchestrating metabolic adaptations to NEB while simultaneously contributing to ketosis pathogenesis. During calving stress and the subsequent lactation period, major shifts occur in key metabolic hormones [46]. Insulin concentrations decline significantly due to reduced glucose availability and increased counter-regulatory hormone activity, while glucagon secretion increases proportionally [47]. This inverse insulin-to-glucagon ratio creates a catabolic hormonal environment that potently stimulates adipose tissue lipolysis and enhances hepatic ketogenesis, thereby amplifying NEFA mobilization and ketone body production [48].

Furthermore, lactating dairy cows frequently develop a state of growth hormone (GH) resistance, wherein target tissues become refractory to GH signaling despite elevated circulating GH concentrations [49]. This uncoupling of the GH-insulin-like growth factor 1 (IGF-1) axis results in decreased IGF-1 levels, which further disrupts glucose homeostasis and impairs insulin sensitivity [50]. The resulting metabolic dysregulation creates a self-perpetuating cycle: reduced insulin action fails to suppress lipolysis adequately, leading to continued NEFA release, hepatic lipid overload, and sustained ketogenesis. Additionally, alterations in other endocrine factors, including cortisol elevation during calving stress and leptin resistance in adipose tissue, contribute to the complex hormonal imbalance that characterizes periparturient metabolic dysfunction and predisposes cows to ketosis.

### 2.4. Oxidative Stress and Inflammatory Responses

Beyond the classical metabolic and hormonal mechanisms, accumulating evidence indicates that oxidative stress and low-grade systemic inflammation represent critical pathophysiological contributors to ketosis development and progression [51,52]. The massive influx of NEFAs and accumulation of ketone bodies impose severe metabolic stress on hepatocytes, disrupting mitochondrial integrity and function. Overwhelmed mitochondria generate excessive reactive oxygen species (ROS), creating an oxidative environment that exceeds the hepatocyte’s antioxidant capacity [53]. This oxidative stress depletes critical antioxidant defense systems, including glutathione peroxidase (GSH-Px), superoxide dismutase (SOD), and catalase, rendering hepatocytes vulnerable to oxidative damage and cellular dysfunction [1,2].

Concurrently, both elevated NEFAs and BHB function as metabolic danger signals that activate pro-inflammatory signaling cascades [1,4]. Specifically, these metabolites trigger activation of nuclear factor kappa-light-chain-enhancer of activated B cells (NF-κB), a master transcriptional regulator of inflammatory responses. NF-κB activation initiates expression of pro-inflammatory cytokines such as tumor necrosis factor-alpha (TNF-α), interleukin-1β (IL-1β), and interleukin-6 (IL-6), establishing a state of chronic low-grade inflammation [1,4]. This inflammatory milieu further exacerbates insulin resistance, impairs hepatic function, and compromises immune competence, creating a vicious cycle that links metabolic stress with immune dysregulation in ketotic cows. Moreover, the convergence of oxidative stress and inflammation damages hepatocyte membranes, mitochondria, and DNA, potentially triggering apoptotic pathways that worsen liver dysfunction and metabolic decompensation [54]. This multifactorial interplay among oxidative damage, inflammation, and metabolic dysfunction underscores the complex pathophysiology of bovine ketosis and highlights the need for comprehensive therapeutic approaches targeting multiple pathogenic mechanisms simultaneously. Over the past decade, the integration of genomic technologies with traditional phenotypic evaluation has transformed our understanding of ketosis from a purely nutritional and management problem to a heritable trait with identifiable genetic determinants.

## 3. Potential Candidate Genes Associated with Ketosis in Dairy Cows

The pathophysiological mechanisms of ketosis, including negative energy balance, hepatic lipid overload, ketogenesis, hormonal dysregulation, and oxidative stress, represent complex metabolic processes that are fundamentally governed by the combined effects of genetic architecture and molecular regulation. The substantial individual variation observed in ketosis susceptibility among dairy cows under similar management conditions strongly suggests an underlying genetic component to disease predisposition. Recent advances in genomic technologies, including genome-wide association studies (GWAS), RNA sequencing, and functional genomics, have revolutionized our understanding of the molecular basis of bovine ketosis. These approaches have facilitated the identification of candidate genes and genetic variants that influence multiple aspects of ketosis pathogenesis, from lipid metabolism and ketone body synthesis to hormonal signaling and cellular stress responses. Elucidating these genetic determinants not only enhances our mechanistic understanding of disease etiology but also provides valuable tools for selective breeding programs aimed at improving metabolic resilience in dairy cattle.

### 3.1. Genes Governing Lipid Mobilization, Synthesis, and Oxidation

Ketosis is intrinsically linked to profound disturbances in energy metabolism during early lactation, predominantly characterized by enhanced adipose tissue lipolysis, excessive hepatic ketogenesis, and impaired peripheral glucose utilization [51]. At the molecular level, several key genes orchestrate fatty acid activation, intracellular transport, and oxidative catabolism, thereby playing pivotal roles in determining the magnitude of lipid mobilization and subsequent ketone body formation.

Among the most critical regulators of fatty acid metabolism is ACSL1 (long-chain acyl-CoA synthetase 1), which catalyzes the activation of long-chain fatty acids to their corresponding Acyl-CoA derivatives, a prerequisite step for both β-oxidation and complex lipid synthesis [55]. Similarly, CPT1A (carnitine palmitoyltransferase 1A) and CPT2 (carnitine palmitoyltransferase 2) constitute essential components of the carnitine shuttle system, facilitating the transport of long-chain fatty acyl-CoA molecules across the mitochondrial membrane for subsequent β-oxidation [56,57,58,59,60]. Furthermore, ACOX1 (Acyl-CoA oxidase 1) and ACAA2 (3-ketoacyl-CoA thiolase 2) participate in peroxisomal and mitochondrial fatty acid oxidation pathways, respectively, contributing to the complete catabolism of fatty acids [61]. Transcriptomic analyses have consistently demonstrated that these genes are significantly upregulated in ketotic cows compared to healthy counterparts, reflecting the heightened lipid catabolic activity characteristic of the ketotic state.

Beyond these core metabolic enzymes, genetic polymorphism studies have identified specific variants associated with differential ketosis susceptibility. Notably, investigations have revealed that the Osteopontin (SPP1) c.495C>T polymorphism located on chromosome 6 is significantly associated with reduced ketosis risk, suggesting that genetic variation in Osteopontin—a multifunctional glycoprotein involved in immune regulation and cellular signaling—may influence metabolic resilience during the transition period [56]. The g.-572 A>G single-nucleotide polymorphism (SNP) within the APOA1 gene promoter in Chinese Holstein cows is associated with enhanced resistance to ketosis, with the GG genotype exhibiting lower circulating BHB concentrations and higher APOA1 transcriptional activity [62]. Additionally, genes encoding fatty acid binding proteins, particularly FABP1 (Fatty acid binding protein 1), which facilitates intracellular fatty acid trafficking and metabolic channeling, exhibit altered expression patterns in ketotic animals [57]. Transcription factors governing lipid metabolism, including PPARα (peroxisome proliferator-activated receptor alpha), which promotes fatty acid oxidation, and SREBP1 (sterol regulatory element-binding protein 1), which orchestrates lipogenic gene expression, along with their downstream targets such as ACC1 (Acetyl-CoA carboxylase 1), FAS (Fatty acid synthase), and SCD1 (Stearoyl-CoA desaturase 1), show significant differential expression between healthy and ketotic cows [57]. These findings underscore the complex transcriptional reprogramming that accompanies metabolic adaptation to NEB.

GWAS combined with fine-mapping approaches have further expanded the catalog of genetic loci implicated in ketosis susceptibility. These comprehensive genomic screens have identified quantitative trait loci (QTL) harboring candidate genes such as *LOC783947*, *DGAT1* (*Diacylglycerol O-acyltransferase 1*), and *LOC107133096*, all of which demonstrate significant associations with ketosis-related phenotypes [58,63]. The *DGAT1* gene, which encodes the terminal enzyme in triglyceride synthesis, is particularly noteworthy given its dual role in hepatic lipid storage and milk fat production, thereby positioning it at the metabolic crossroads of energy partitioning in lactating dairy cows [64].

Moreover, genes mediating cellular responses to metabolic stress have emerged as important modulators of ketosis pathogenesis. SOD2 (Superoxide dismutase 2), a mitochondrial antioxidant enzyme, and HIF-2α (Hypoxia-inducible factor 2 alpha), a transcription factor responsive to cellular stress conditions, both participate in oxidative stress responses and show altered expression patterns in ketotic animals [65]. Additionally, CD36 (cluster of differentiation 36), a scavenger receptor facilitating NEFA uptake into hepatocytes, represents a critical determinant of hepatic lipid influx. Several studies have demonstrated that overexpression of CD36 exacerbates NEFA-induced hepatic lipotoxicity and lipid accumulation, thereby amplifying the pathological cascade leading to fatty liver and ketosis [65,66,67]. Collectively, these genes constitute an integrated molecular network that governs lipid mobilization, hepatic uptake, oxidative metabolism, and cellular stress responses, ultimately determining individual susceptibility to ketosis during the periparturient period.

### 3.2. Genes Central to Ketone Body Metabolism and Energy Homeostasis

While lipid mobilization and oxidation provide the metabolic substrates for ketogenesis, the actual synthesis and utilization of ketone bodies are governed by a discrete set of enzymes that constitute the core ketogenic and ketolytic machinery. Understanding the genetic regulation of these enzymes is essential for comprehending the biochemical basis of hyperketonemia and identifying potential therapeutic targets.

The ketogenic pathway is orchestrated by several sequential enzymatic reactions, each catalyzed by specific gene products. ACAT1, also known as mitochondrial thiolase, initiates ketogenesis by catalyzing the condensation of two Acetyl-CoA molecules to form Acetoacetyl-CoA, thereby providing the foundational substrate for subsequent ketogenic reactions [68]. Following this initial condensation, HMGCS2 (3-hydroxy-3-methylglutaryl-CoA synthase 2) functions as the rate-limiting enzyme of ketogenesis, mediating the condensation of acetoacetyl-CoA with another acetyl-CoA molecule to generate 3-hydroxy-3-methylglutaryl-CoA (HMG-CoA) [69]. Subsequently, HMGCL (HMG-CoA lyase) catalyzes the cleavage of HMG-CoA to yield acetoacetate and acetyl-CoA, completing the core synthetic sequence [70]. The resulting acetoacetate serves as the immediate precursor for the two major circulating ketone bodies: it can be reversibly reduced to β-hydroxybutyrate (BHB) through the action of BDH1 (β-hydroxybutyrate dehydrogenase 1), or it can undergo spontaneous non-enzymatic decarboxylation to produce acetone [71]. The relative activities of these enzymes, particularly the reversible reaction catalyzed by BDH1, determine the ratio of acetoacetate to BHB in circulation, with BHB typically predominating due to the hepatic redox state favoring its formation [71].

While hepatic ketogenesis represents the source of circulating ketone bodies, peripheral tissue utilization constitutes the sink that normally maintains ketone body homeostasis. OXCT1 (3-oxoacid CoA-transferase 1), also known as succinyl-CoA:3-ketoacid coenzyme A transferase (SCOT), serves as the rate-limiting enzyme for ketone body utilization in extra-hepatic tissues such as skeletal muscle, cardiac muscle, and brain [33]. This enzyme catalyzes the transfer of coenzyme A from succinyl-CoA to acetoacetate, thereby activating acetoacetate for entry into the tricarboxylic acid (TCA) cycle and subsequent oxidative metabolism [72]. Importantly, hepatocytes lack OXCT1 expression, rendering the liver incapable of utilizing the ketone bodies it produces—a metabolic specialization that ensures hepatic ketones are exported exclusively for peripheral tissue consumption [73]. Collectively, these genes, including ACAT1, HMGCS2, HMGCL, BDH1, and OXCT1, form an integrated regulatory network governing hepatic ketone body synthesis, interconversion, and peripheral utilization, thereby maintaining systemic energy balance during periods of metabolic stress. Dysregulation of this network, particularly through excessive upregulation of hepatic ketogenic enzymes or inadequate peripheral ketolytic capacity, underlies the pathological accumulation of ketone bodies characteristic of clinical and subclinical ketosis.

### 3.3. Genes Regulating Hormonal Signaling and Metabolic Transcription

Beyond the direct metabolic enzymes discussed above, ketosis pathogenesis is profoundly influenced by genes encoding transcription factors and signaling molecules that coordinate whole-body energy metabolism and metabolic adaptation to negative energy balance. These regulatory genes integrate nutritional, hormonal, and metabolic signals to orchestrate appropriate transcriptional responses in lipid and carbohydrate metabolism.

Central among these regulatory factors are the nuclear receptors PPARα (peroxisome proliferator-activated receptor alpha) and SREBF1 (sterol regulatory element-binding transcription factor 1, also known as SREBP1), which serve as master transcriptional coordinators of fatty acid oxidation and lipid synthesis, respectively [70,74]. PPARα, activated by fatty acids and their derivatives, promotes the transcription of genes involved in fatty acid uptake, mitochondrial and peroxisomal β-oxidation, and ketogenesis, thereby facilitating the metabolic adaptation to lipid influx during negative energy balance [75]. Conversely, SREBF1 stimulates the expression of lipogenic enzymes, and its dysregulation in ketotic cows contributes to the paradoxical continuation of hepatic lipid synthesis despite massive lipid influx, exacerbating hepatic triglyceride accumulation [59]. The balance between these opposing transcriptional programs—catabolic fatty acid oxidation driven by PPARα and anabolic lipogenesis promoted by SREBF1—is critical in determining whether hepatic lipid metabolism remains adaptive or progresses to pathological fatty liver and ketosis.

Hormonal signaling pathways, particularly those involving insulin and its downstream effectors, constitute another crucial layer of genetic regulation in ketosis pathogenesis. Alterations in the expression or function of insulin-related genes, including INSR (insulin receptor), IRS1 (insulin receptor substrate 1), and AKT1 (AKT serine/threonine kinase 1), profoundly influence insulin sensitivity, glucose uptake, and the regulation of adipose tissue lipolysis [59]. During the periparturient period, reduced insulin signaling—whether due to decreased insulin secretion, impaired receptor function, or downstream signaling defects—fails to adequately suppress hormone-sensitive lipase in adipocytes, thereby perpetuating excessive fat mobilization and NEFA release [76]. Furthermore, recent investigations have revealed that elevated expression of MAPK1 (mitogen-activated protein kinase 1) in response to elevated BHB concentrations contributes to hepatic lipotoxicity and metabolic dysfunction [59]. Specifically, BHB-induced activation of MAPK1 signaling promotes pro-inflammatory responses and cellular stress pathways in hepatocytes, creating a self-reinforcing cycle wherein ketone bodies themselves exacerbate hepatic dysfunction and metabolic derangement. These findings highlight the complex interplay between metabolic substrates and cellular signaling cascades in ketosis pathogenesis, emphasizing that ketone bodies function not merely as energy metabolites but also as bioactive signaling molecules capable of modulating gene expression and cellular function.

### 3.4. Emerging Molecular Regulators and Multi-Omics Discovery of Ketosis-Associated Genes

The advent of high-throughput genomic technologies and integrative multi-omics approaches has dramatically accelerated the discovery of novel genes and regulatory networks implicated in bovine ketosis, revealing a complexity far exceeding the classical metabolic pathways previously described. These contemporary investigations have employed diverse methodologies, including GWAS, transcriptomics, metabolomics, and weighted gene co-expression network analysis to systematically identify genetic determinants of ketosis susceptibility and elucidate their functional roles in disease pathogenesis.

Early genomic investigations identified multiple SNPs located on bovine chromosomes 14, 20, and 27 that exhibited significant associations with blood BHB concentrations in early-lactation Holstein cows, establishing that genetic variation at these loci influences ketone body metabolism and ketosis risk [77]. Building upon these foundational observations, Li and coworkers employed an innovative metabolomics-genomics integration strategy to validate the central regulatory roles of several key genes, including DGAT1, ACSL1, HMGCS2, OXCT1, and PPARGC1A (peroxisome proliferator-activated receptor gamma coactivator 1-alpha), in orchestrating energy metabolism during the transition period. This integrative approach, which correlates genetic variation with metabolite profiles, provides powerful evidence linking specific genes to their functional metabolic consequences.

Expanding the genetic landscape further, comprehensive GWAS analyses have identified multiple genes demonstrating genome-wide significant associations with blood BHB concentrations [60]. Among these discoveries were genes intimately involved in insulin regulation, such as INSIG2 (insulin-induced gene 2), which modulates cholesterol and fatty acid synthesis in response to insulin signaling, as well as genes directly participating in fatty acid metabolism, including HADHB (hydroxyacyl-CoA dehydrogenase trosine B), HADHA (hydroxyacyl-CoA dehydrogenase trosine A), and PANK2 (pantothenate kinase 2) [60]. The *HADHA* and *HADHB* genes encode subunits of the mitochondrial trifunctional protein, which catalyzes three sequential steps in the β-oxidation of long-chain fatty acids, thereby directly influencing the substrate flux toward ketogenesis [71]. Meanwhile, PANK2 participates in coenzyme A biosynthesis, providing an essential cofactor for fatty acid metabolism and ketogenesis [78].

Complementing these association-based approaches, systems biology methodologies have revealed higher-order regulatory relationships among genes involved in ketosis. Through weighted gene co-expression network analysis (WGCNA), ACACA (acetyl-CoA carboxylase alpha), ELOVL6 (elongation of very long chain fatty acids protein 6), and XPO7 (exportin 7) were identified as key regulators of low-intake-induced ketosis in dairy cows [79]. These genes appear to function as network hubs, coordinating the expression of multiple downstream targets involved in lipid metabolism and metabolic adaptation. Concurrently, multiple novel QTL regions were significantly enriched with regulatory elements associated with fatty acid metabolism and insulin signaling pathways, suggesting that genetic variations in these regions influence ketosis susceptibility through effects on gene regulatory networks rather than structural protein changes alone [80].

Multi-omics integration studies have proven particularly valuable for identifying differentially expressed genes that distinguish ketotic from healthy cows. Using a comprehensive multi-omics analysis integrating transcriptomic, proteomic, and metabolomic data, 14 differentially expressed genes were identified—namely ALB (albumin), CPT1A, PC (pyruvate carboxylase), UGT2B10 (UDP glucuronosyltransferase family 2 member B10), SHROOM3, FN1 (fibronectin 1), G3BP2 (G3BP stress granule assembly factor 2), ANXA3 (annexin A3), PTK2 (protein tyrosine kinase 2), ACSL1, BIRC6 (baculoviral IAP repeat containing 6), CSN1S1 (casein alpha S1), HAO2 (hydroxyacid oxidase 2), and RASSF6 (Ras association domain family member 6)—that exhibited consistent alterations across multiple molecular levels in ketotic animals [81]. Among these, ACSL1 emerged as particularly significant: this enzyme activates long-chain fatty acids to their acyl-CoA derivatives, thereby enabling their transmembrane transport and providing substrates for both β-oxidation and complex lipid biosynthesis [82]. The functional importance of ACSL1 is underscored by genetic studies in mice demonstrating that hepatic ACSL1 deficiency severely impairs fatty acid oxidation not only in the liver but also in peripheral tissues such as muscle and adipose tissue, highlighting its systemic metabolic importance [83].

Recent investigations employing integrated RNA sequencing and GWAS association analysis in Holstein dairy cows have identified additional ketosis candidate genes with previously unrecognized metabolic roles. Through comprehensive transcriptomic profiling coupled with genetic association testing in 12 Holstein dairy cows, five novel ketosis candidate genes were identified, including MAFA (MAF bZIP transcription factor A), C14H8orf82 (chromosome 14 H8orf82 homolog), MAF1 (MAF1 homolog, negative regulator of RNA polymerase III), GRINA (glutamate receptor, ionotropic, N-methyl D-aspartate-associated protein), and RECQL4 (RecQ like helicase 4) [60]. To validate the relevance of these candidates across species and disease contexts, the investigators conducted phenome-wide association studies (Phe-WAS) examining orthologous human genes across multiple complex traits and diseases, confirming their critical involvement in metabolic and immunological processes [84]. This cross-species validation approach strengthens the biological plausibility of these candidates and suggests that fundamental mechanisms of metabolic regulation are evolutionarily conserved between cattle and humans.

Further GWAS investigations have pinpointed specific chromosomal regions harboring ketosis-associated variants. Through systematic genome-wide screening, researchers identified five candidate SNP loci distributed across bovine chromosomes 5, 8, 9, and 15 that demonstrated significant associations with ketone metabolism phenotypes [85]. The most strongly associated SNP, rs109896020, maps to chromosome 5, and within the genomic interval surrounding this variant, four potential candidate genes were identified: EFCAB6 (EF-hand calcium binding domain 6), PARVB (Parvin beta), PARVGSHISAL1, and KIAA1644. Notably, PARVB has been previously implicated in non-alcoholic fatty liver disease in humans and is positioned in close genomic proximity (61,382 base pairs) to SNP rs109896020, suggesting it may represent the causal gene underlying this association signal [81]. The identification of PARVB links genetic variation in cell adhesion and cytoskeletal organization to metabolic liver disease, revealing unexpected biological connections between cellular structure and metabolic function.

Beyond genomic and transcriptomic discoveries, recent mechanistic investigations have unveiled novel regulatory factors governing adipose tissue metabolism during ketosis. Research examining SCK has revealed that adipose tissue in affected cows undergoes excessive lipolysis that is closely associated with enhanced activity of transcription factor EB (TFEB), a master regulator of autophagy and lysosomal biogenesis [82]. Specifically, marked translocation of TFEB from the cytoplasm to the nucleus was observed in adipocytes from SCK cows, and subsequent in vitro experiments confirmed that increased TFEB transcriptional activity promotes lipolysis in adipocytes through upregulation of lipase genes, while genetic or pharmacological inhibition of TFEB attenuates lipolysis [82]. This discovery identifies TFEB as a novel therapeutic target for modulating adipose tissue fat mobilization during negative energy balance.

Additionally, through systematic mechanistic investigations, NDUFAB1 (NADH: ubiquinone oxidoreductase subunit AB1) was identified for the first time as a core metabolic regulator whose activation effectively mitigates the cytotoxic damage caused by NEFA to adipocytes [83]. NDUFAB1 encodes a component of mitochondrial complex I of the electron transport chain, and its protective effects appear to involve enhancement of mitochondrial function and reduction in NEFA-induced oxidative stress in adipocytes [83]. This finding highlights the critical importance of maintaining mitochondrial integrity in adipose tissue for metabolic health during the transition period.

Collectively, these emerging discoveries from diverse genomic and molecular approaches have substantially expanded our understanding of ketosis genetics, revealing that disease susceptibility is influenced not only by classical metabolic genes but also by regulatory factors controlling gene expression networks, cellular stress responses, organellar function, and inter-tissue metabolic communication. This expanded genetic architecture provides a comprehensive foundation for future mechanistic investigations and genomic selection strategies.

### 3.5. Signaling Pathways Integrating Metabolic Stress and Cellular Responses in Ketosis

The onset and progression of ketosis cannot be attributed to isolated enzymatic deficiencies or single-gene mutations; rather, it represents a systemic metabolic derangement arising from dysregulation of complex and interconnected cellular signaling networks activated by NEFA lipotoxicity and metabolic stress. These signaling pathways integrate nutrient sensing, energy homeostasis, oxidative stress responses, and cellular adaptation mechanisms, collectively determining whether metabolic adaptation remains physiological or progresses to pathological ketosis. Understanding these signaling cascades is essential for comprehending disease pathogenesis at the systems level and identifying potential therapeutic intervention points.

The AMP-activated protein kinase (AMPK) signaling pathway functions as a central energy sensor and metabolic regulator, coordinating cellular responses to energetic stress [85]. Under conditions of cellular energy depletion, characterized by elevated AMP:ATP or ADP:ATP ratios, AMPK undergoes allosteric activation and phosphorylation, triggering a metabolic switch that promotes catabolic processes while inhibiting anabolic pathways [86]. Specifically, activated AMPK stimulates fatty acid oxidation by phosphorylating and inactivating acetyl-CoA carboxylase, thereby reducing malonyl-CoA levels and relieving the inhibition of carnitine palmitoyltransferase 1 (CPT1), the rate-limiting enzyme for mitochondrial fatty acid import [86]. Consequently, AMPK activation during energy deficiency enhances β-oxidation, thereby supplying abundant acetyl-CoA substrates for ketone body production in hepatocytes [85]. While this adaptive response serves to generate alternative fuels for peripheral tissues, excessive or prolonged AMPK activation can contribute to pathological ketogenesis when fatty acid oxidation overwhelms ketone body utilization.

The peroxisome proliferator-activated receptor alpha (PPARα) signaling pathway serves as the master transcriptional regulator of hepatic fatty acid metabolism and ketogenesis. PPARα, activated by fatty acids and their derivatives, forms heterodimers with retinoid X receptor (RXR) and binds to peroxisome proliferator response elements (PPREs) in the promoter regions of target genes, thereby inducing their transcription [87]. In the liver, PPARα directly modulates the expression of key ketogenic enzymes, including HMGCS2, BDH1, and ACAT1, as well as genes involved in fatty acid uptake and oxidation [88]. During negative energy balance, when circulating NEFA concentrations rise dramatically, hepatic PPARα becomes highly activated, driving coordinated upregulation of the entire ketogenic program [89]. While this transcriptional response represents an adaptive mechanism to metabolize excess fatty acids and generate alternative fuels, enhanced PPARα activity simultaneously promotes excessive ketone body production, thereby contributing to hyperketonemia [90]. The central role of PPARα in orchestrating ketogenesis has been conclusively demonstrated in PPARα-knockout mice, which exhibit severely impaired ketogenic capacity and develop hepatic steatosis when challenged with fasting or high-fat diets.

The mechanistic target of rapamycin (mTOR) signaling pathway integrates nutrient availability, growth factor signaling, and cellular energy status to regulate anabolic processes, protein synthesis, and autophagy [91]. Under nutrient-replete conditions, mTOR complex 1 (mTORC1) is activated and promotes cellular growth, proliferation, and biosynthetic pathways while simultaneously inhibiting autophagy [91]. However, during energy deficiency characteristic of early lactation, declining nutrient availability and reduced insulin/IGF-1 signaling result in mTOR suppression [92]. Consequently, mTORC1 inactivation relieves the inhibition of autophagy, thereby promoting cellular self-digestion and lipolysis to liberate amino acids and fatty acids that can serve as substrates for gluconeogenesis and ketone body synthesis [92]. Additionally, mTOR suppression enhances the activity of transcription factors such as FoxO and PPARα, further amplifying catabolic pathways including ketogenesis. Thus, the mTOR pathway functions as a critical metabolic switch, with its inactivation during negative energy balance contributing to the metabolic milieu conducive to ketosis development.

The forkhead box O (FoxO) family of transcription factors, particularly FoxO1, plays essential roles in energy stress responses, gluconeogenesis, and metabolic adaptation to fasting [93]. During insulin deficiency or insulin resistance, hallmark features of early lactation and negative energy balance, FoxO transcription factors fail to be phosphorylated by AKT, enabling their translocation from the cytoplasm to the nucleus, where they drive the transcription of target genes [93]. In hepatocytes, nuclear FoxO1 promotes the expression of key gluconeogenic enzymes, including phosphoenolpyruvate carboxykinase (PEPCK) and glucose-6-phosphatase (G6PC), thereby enhancing hepatic glucose production to support milk lactose synthesis and maintain blood glucose homeostasis [93]. However, excessive or dysregulated FoxO1 activation can lead to energy metabolism disorders and contribute to increased ketone body production through several mechanisms: first, by depleting oxaloacetate through its diversion to gluconeogenesis, FoxO1 activation reduces oxaloacetate availability for citric acid cycle function, thereby shunting excess acetyl-CoA toward ketogenesis; second, FoxO1 may directly or indirectly influence the expression of ketogenic enzymes. Consequently, while FoxO signaling serves adaptive functions during energy scarcity, its dysregulation contributes to the metabolic derangements underlying ketosis.

Beyond these nutrient-sensing pathways, oxidative stress-induced signaling cascades play critical pathogenic roles in ketosis, particularly through the reactive oxygen species (ROS)-p38 MAPK-p53/Nrf2 signaling axis. High concentrations of BHB—the predominant ketone body in bovine ketosis—induce substantial oxidative stress and apoptosis in bovine hepatocytes by activating this complex signaling network [94,95]. Mechanistically, elevated BHB levels promote ROS generation through impaired mitochondrial function, and these ROS serve as signaling molecules that activate p38 mitogen-activated protein kinase (MAPK) through oxidative modification and upstream kinase activation [95,96]. Activated p38 MAPK subsequently phosphorylates and stabilizes p53, a master pro-apoptotic transcription factor, leading to increased p53 expression, enhanced nuclear localization, and elevated transcriptional activity in hepatocytes [84,85,86]. Concurrently, the ROS-p38 signaling axis suppresses the expression and nuclear translocation of nuclear factor erythroid 2-related factor 2 (Nrf2), the master transcriptional activator of cellular antioxidant defense genes [91,92,93]. The reduction in Nrf2 activity diminishes the expression of cytoprotective genes encoding antioxidant enzymes and detoxification proteins, thereby increasing hepatocyte vulnerability to oxidative damage [95,96]. As a consequence of p53 activation and Nrf2 suppression, pro-apoptotic gene expression becomes significantly elevated, culminating in programmed cell death of bovine hepatocytes [95]. This oxidative stress–apoptosis cascade not only impairs hepatic function but also exacerbates metabolic dysfunction, creating a vicious cycle wherein ketone body accumulation promotes cellular damage that further compromises hepatic metabolic capacity.

Recent investigations have additionally identified an imbalance in the fibroblast growth factor 21 (FGF21) and MAPK1 signaling axes as key determinants of hepatic lipid metabolism disorders during ketosis. FGF21, an endocrine hormone secreted primarily by the liver during fasting and metabolic stress, functions to enhance fatty acid oxidation, improve insulin sensitivity, and regulate systemic energy homeostasis [97,98]. However, during ketosis, dysregulation of the FGF21-MAPK1 axis disrupts normal metabolic control: elevated BHB concentrations induce aberrant MAPK1 (also known as ERK2) activation, which promotes hepatic lipid accumulation and lipotoxicity [97]. Experimental interventions have demonstrated that genetic or pharmacological knockdown of MAPK1 effectively reverses BHB-induced lipid accumulation in hepatocytes, while supplementation with recombinant FGF21 restores metabolic balance and ameliorates hepatic steatosis [97]. These findings identify the FGF21-MAPK1 axis as a critical regulatory node linking ketone body accumulation to hepatic lipid metabolism disorders, and suggest that modulation of this pathway may offer therapeutic potential.

The protein kinase RNA-like endoplasmic reticulum kinase (PERK)-eukaryotic initiation factor 2α (eIF2α) signaling pathway represents an adaptive response to endoplasmic reticulum (ER) stress induced by NEFA accumulation in hepatocytes. Mechanistic studies have elucidated that NEFA-induced activation of the PERK-eIF2α pathway exerts complex and seemingly paradoxical effects on hepatic lipid metabolism [99]. On one hand, PERK-eIF2α signaling promotes both lipogenesis and lipid oxidation—the latter serving to generate ATP and thereby provide energy to support cellular function under metabolic stress [99]. On the other hand, this pathway simultaneously inhibits the assembly and secretion of very-low-density lipoproteins (VLDLs), the primary mechanism by which hepatocytes export triglycerides into circulation [99]. Consequently, although enhanced lipid oxidation occurs, the inability to export triglycerides ultimately leads to their progressive accumulation within hepatocytes, manifesting as hepatic steatosis [99]. Importantly, this suggests that activation of the PERK-eIF2α pathway plays a dual role in periparturient dairy cow livers: while it represents an adaptive mechanism enabling hepatocytes to resist NEFA-induced lipotoxicity by enhancing oxidative capacity and helping to alleviate negative energy balance, it simultaneously contributes to fatty liver pathogenesis by blocking lipid export. This example illustrates the complex nature of metabolic adaptation, wherein signaling pathways can be simultaneously protective and pathogenic depending on the physiological context.

Furthermore, high concentrations of NEFA directly activate the PPARα signaling pathway in hepatocytes, significantly accelerating hepatic fatty acid oxidation and consequently producing excess acetyl-CoA, which serves as the primary driving force for ketone body production [100,101,102]. This mechanism directly links the magnitude of fat mobilization to the severity of ketogenesis, explaining why excessive lipolysis invariably leads to hyperketonemia when peripheral ketone body utilization is insufficient [103,104].

Beyond hepatocytes, signaling pathways in other tissues contribute to the ketosis pathophysiology. Notably, mitochondrial deacetylase Sirtuin 3 (SIRT3) has been shown to significantly mitigate oxidative stress-induced apoptosis in bovine mammary epithelial cells by activating the AMPK signaling pathway [105,106,107]. This protective mechanism helps counteract the negative effects of ketosis on mammary gland function, potentially preserving milk production capacity [105]. The discovery that SIRT3-AMPK signaling protects mammary epithelial cells highlights the importance of inter-tissue communication and systemic metabolic coordination in determining the overall impact of ketosis on dairy cow health and productivity [108,109].

In summary, ketosis pathogenesis involves the coordinated dysregulation of multiple interconnected signaling networks—including AMPK, PPARα, mTOR, FoxO, PERK-eIF2α, ROS-p38-p53/Nrf2, and FGF21-MAPK1 pathways—that collectively orchestrate cellular and systemic metabolic responses to negative energy balance and NEFA lipotoxicity. Understanding these complex signaling interactions provides essential mechanistic insights into disease pathophysiology and reveals potential therapeutic targets for preventing or ameliorating ketosis in dairy cattle (Table 1).

## 4. Metabolomic Signatures of Dairy Cow Ketosis

Metabolomics has become an essential approach for elucidating the pathophysiology of periparturient ketosis in dairy cows, identifying early diagnostic biomarkers, and evaluating the metabolic consequences of NEB. Using LC–MS, GC–MS, and ^1^H-NMR platforms, numerous studies have consistently demonstrated not only the classical alterations in ketone body metabolism but also coordinated disturbances across amino acid, lipid, and carbohydrate metabolic pathways.

### 4.1. Blood (Serum/Plasma): Core Systemic Metabolic Disturbances

Blood (serum or plasma) offers the most direct and representative snapshot of systemic metabolic status [116]. Classical hallmarks of ketosis include marked elevations in BHBA, acetoacetate, and acetone [16]. Concurrently, intense adipose mobilization results in substantial increases in NEFA and long-chain fatty acids such as palmitic and stearic acids [17]. GC–MS studies have specifically identified elevated cis-9-hexadecenoic acid, proposing it as a potential candidate biomarker for clinical ketosis [117]. Amino acid metabolism is also profoundly altered: Concentrations of glucogenic amino acids—including alanine, glutamate, glutamine, histidine, and phenylalanine—typically decline due to increased hepatic gluconeogenic demand, while altered lysine catabolism is reflected by elevated 2-piperidinecarboxylic acid [117,118]. Furthermore, disturbances in acylcarnitine profiles (short-and medium-chain acylcarnitines) link ketosis with impaired mitochondrial β-oxidation [119]. Disturbances extend to the gut-microbial axis, evidenced by increased levels of kynurenine and the uremic toxin 3-indoxyl sulfate, metabolites of tryptophan metabolism, which point toward systemic inflammation and potential renal toxicity. In addition, decreased concentrations of antioxidant-related amino acids like taurine, glycine, and proline have been observed in ketotic water buffalo [120].

### 4.2. Urine: Noninvasive Monitoring and Excretory Signatures

Urine metabolomics reveals downstream excretion patterns and provides a noninvasive window into whole-body metabolism [121]. Similar to serum, urinary BHBA and acetone are consistently elevated in ketotic cows. Metabolic pathway enrichment analyses often show significant alterations in phenylalanine metabolism as well as the alanine, aspartate, and glutamate pathways, underscoring the close correspondence between urinary and systemic metabolic fingerprints [122]. A notable finding is the reduction in urinary hippuric acid and TMAO (trimethylamine N-oxide) in cows with concurrent abomasal displacement (DA) and ketosis, which is strongly associated with decreased feed intake and rumen microbial dysbiosis affecting the synthesis and clearance of gut-derived metabolites [123].

### 4.3. Milk: Mammary Function and Herd-Level Surveillance

Milk metabolomics provides an efficient, high-throughput means for herd-level surveillance, as milk composition directly reflects mammary gland metabolism and systemic energy status. Milk BHBA is strongly correlated with blood BHBA and is a robust NEB indicator. Metabolites such as glycine and acetylcarnitine exhibit patterns consistent with the severity of NEB, reflecting altered fatty acid transport and utilization by the mammary gland [124]. Furthermore, decreased concentrations of intermediates involved in purine and pyrimidine metabolism—such as galactose-1-phosphate, glucose-1-phosphate, uridine, and orotate—have been reported in milk from ketotic cows, suggesting potential impairments in mammary epithelial cell turnover and nucleotide biosynthesis [125]. Changes in key TCA cycle-related metabolites like citrate and formate are also observed. Mid-infrared (MIR/FTIR) spectroscopy enhances applied monitoring by enabling high-throughput prediction of milk BHBA and acetone, alongside estimates of circulating NEFA and urea, providing a cost-effective strategy for large-scale metabolic risk screening [125].

### 4.4. Tissue and Rumen Fluid: Pathogenesis and Local Metabolic Impairments

Tissue-level metabolomics provides critical, site-specific insights into the downstream consequences of ketosis. Skeletal muscle, a major site of ketone utilization, exhibits suppressed TCA cycle activity during clinical ketosis. GC-MS/Proteomics analyses reveal reduced levels of key TCA intermediates, including pyruvate, succinate, α-ketoglutarate, fumarate, malate, and citrate, alongside increased local BHBA accumulation, indicating constrained mitochondrial oxidation and ATP production [126]. In the liver, ketosis is consistently associated with features indicative of fatty liver disease (FLD), characterized by the accumulation of lipids and cholesterol, and reduced hepatic glucose concentrations [119]. Multi-channel analysis has identified novel FLD biomarkers, including increased glycocholate and tauroursodeoxycholate (bile acids), and specific lipid molecules such as cholesteryl linoleate [127]. In adipose tissue, multi-omics analyses reveal that SCK disrupts immunometabolic homeostasis by perturbing the sphingolipid metabolic pathway, notably leading to the accumulation of ceramides, which are implicated in oxidative stress and inflammation [127].

As the primary site of volatile fatty acid (VFA) production, the rumen plays an essential upstream role by supplying substrates for hepatic gluconeogenesis. Metabolomics studies of rumen fluid consistently report elevated concentrations of ketogenic VFA—specifically butyrate and valerate—and a significant reduction in propionate, the main gluconeogenic precursor [121]. This adverse shift in VFA ratio directly limits the availability of glucose precursors for the liver, exacerbating systemic NEB and promoting ketone body overproduction [122]. Furthermore, changes in other rumen metabolites like formate and succinate are indicative of shifts in the microbial community and fermentation patterns, highlighting the crucial need to manage rumen health as a core component of ketosis prevention strategies [123].

In summary, metabolomics provides an integrated view of the systemic metabolic disruptions underlying ketosis in dairy cows, revealing consistent alterations in lipid mobilization, ketone body production, amino acid utilization, and rumen fermentation. These multi-matrix metabolic signatures improve our understanding of disease mechanisms and offer valuable candidates for early diagnosis and herd-level monitoring. As analytical technologies advance, metabolomics will continue to strengthen precision detection and targeted management strategies for mitigating ketosis in modern dairy systems.

## 5. Integrated Preventive Strategies for Bovine Ketosis

Given the multifactorial etiology and complex pathophysiology of bovine ketosis elucidated in preceding sections, prevention strategies must adopt a comprehensive, integrated approach that addresses nutritional management, environmental optimization, and metabolic monitoring throughout the periparturient period. Effective ketosis prevention requires proactive intervention beginning well before calving and continuing through early lactation, with particular emphasis on minimizing the severity and duration of negative energy balance. The following subsections delineate evidence-based preventive measures organized according to their primary mechanisms of action and practical implementation.

### 5.1. Nutritional Management During the Transition Period

Nutritional management during the dry period and early lactation represents the cornerstone of ketosis prevention, as dietary composition and feeding strategies directly influence energy balance, metabolic adaptation, and disease susceptibility [128]. The transition period—spanning approximately three weeks before to three weeks after calving—demands particularly meticulous attention to nutritional programming, as this interval encompasses the most dramatic metabolic changes and the highest risk for ketosis development [129]. Strategic nutritional interventions during this critical window can substantially mitigate the severity of negative energy balance and reduce the incidence of both subclinical and clinical ketosis [130].

#### 5.1.1. Body Condition Management and Prepartum Nutrition

A fundamental principle of ketosis prevention centers on maintaining optimal body condition scores throughout the dry period, thereby avoiding excessive adiposity at calving. Research has conclusively demonstrated that overconditioned cows, those with body condition scores exceeding 3.5–4.0 on a five-point scale, face a significantly elevated risk of ketosis compared to cows maintained at a moderate body condition [131]. The mechanistic basis for this association relates to the fact that obese cows experience more severe negative energy balance postpartum due to two compounding factors: first, adipose tissue in overconditioned cows exhibits enhanced lipolytic sensitivity, resulting in disproportionately greater fat mobilization in response to energy deficit; second, excessive body fatness correlates inversely with voluntary feed intake during early lactation, thereby exacerbating the energetic shortfall [132]. Consequently, strategic nutritional management during the far-off dry period (60–21 days before calving) should aim to achieve moderate body condition scores at calving, typically between 3.0 and 3.5 on a five-point scale, through controlled energy intake that prevents excessive fat accumulation while maintaining adequate nutrient reserves for the impending lactation [133].

During the close-up period (the final 21 days before calving), nutritional management objectives shift toward preparing the rumen microbiome and metabolic machinery for the high-concentrate, energy-dense diets characteristic of early lactation. This adaptation period should feature gradual increases in dietary energy density and concentrate inclusion, typically progressing from 30–35% concentrate in far-off dry cow rations to 40–50% concentrate in close-up diets [97]. This progressive dietary transition facilitates rumen papillae development, enhances volatile fatty acid absorption capacity, and promotes metabolic adaptation to increased glucose precursor availability, thereby reducing the metabolic shock associated with abrupt dietary changes at calving.

#### 5.1.2. Environmental Optimization and Stress Reduction

Beyond nutritional interventions, environmental management constitutes a critical yet often underappreciated component of ketosis prevention. The housing environment for transition cows must be meticulously maintained to provide dry, well-ventilated, and comfortable conditions that minimize physiological stress and support optimal immune function [19]. Environmental stressors, including thermal stress, inadequate ventilation, excessive humidity, and uncomfortable lying surfaces, activate stress response pathways that promote cortisol secretion, suppress immune function, reduce feed intake, and exacerbate negative energy balance, thereby collectively elevating ketosis susceptibility [134].

Specifically, several environmental factors warrant careful attention: First, stocking density must be carefully controlled to prevent overcrowding, as excessive competition for feed bunk access and resting space reduces dry matter intake and increases psychological stress [135]. Research indicates that maintaining stocking densities below 80% of pen capacity in transition cow facilities substantially improves feed intake, reduces aggressive interactions, and decreases metabolic disease incidence [89]. Second, thermal stress—whether from excessive heat during summer months or inadequate cold protection during winter—profoundly influences metabolic homeostasis and ketosis risk [136]. Heat stress suppresses voluntary feed intake, diverts metabolic resources toward thermoregulatory mechanisms, and impairs insulin signaling, thereby amplifying negative energy balance and predisposing cows to ketosis [137]. Conversely, cold stress without adequate dietary energy compensation increases maintenance energy requirements and can similarly precipitate negative energy balance. Therefore, environmental management systems must incorporate effective cooling strategies during heat stress periods (shade, fans, evaporative cooling) and adequate wind protection and bedding during cold stress periods to minimize the metabolic burden of thermoregulation [138]. Third, pen design and management should prioritize cow comfort, providing ample bunk space (minimum 30 inches per cow), comfortable lying surfaces with adequate bedding depth, and sufficient space for normal social behaviors, all of which promote optimal feed intake and reduce stress-induced metabolic perturbations [28].

#### 5.1.3. Dietary Composition and Nutrient Balance

The formulation of transition cow diets requires sophisticated balancing of multiple nutritional parameters to simultaneously optimize energy intake, metabolic adaptation, and rumen health while avoiding metabolic disorders. Feed formulations must be scientifically designed with appropriate forage-to-concentrate ratios that support adequate dry matter intake while providing sufficient energy density to minimize negative energy balance [139]. Typically, close-up and fresh cow diets should contain 40–55% concentrate (dry matter basis), although optimal ratios vary depending on forage quality, individual farm management, and cow genetics [140].

Protein nutrition during the transition period demands particular attention, as both deficiency and excess can compromise metabolic health and increase ketosis risk. Dietary crude protein should be carefully controlled to approximately 14–16% of diet dry matter, as excessive protein intake—particularly when it exceeds 17–18% of dry matter—generates substantial metabolic liabilities. Specifically, surplus dietary protein undergoes deamination in the rumen and liver, producing ammonia that must be detoxified to urea through the energetically expensive urea cycle, thereby diverting glucose and ATP from productive purposes [141]. Additionally, excess protein catabolism generates glucogenic amino acids that, paradoxically, increase hepatic gluconeogenic burden and may exacerbate metabolic stress [142]. Furthermore, elevated blood urea nitrogen concentrations resulting from excess protein intake can impair reproductive performance and immune function [143]. Therefore, transition cow diets should provide adequate but not excessive protein, with emphasis on high-quality, digestible protein sources and appropriate amino acid balance, particularly ensuring sufficient metabolizable methionine and lysine to support milk protein synthesis without generating excess nitrogen.

Carbohydrate nutrition represents another critical dietary consideration with profound implications for ketosis prevention. The selection and processing of dietary carbohydrate sources significantly influence glucose precursor availability, rumen fermentation patterns, and metabolic outcomes. Ground corn is particularly recommended as the primary starch source in transition cow diets due to its favorable fermentation characteristics: corn starch undergoes moderate ruminal fermentation that generates propionate—the primary gluconeogenic precursor in ruminants—without producing excessive lactate that could precipitate rumen acidosis [144]. Moreover, appropriately processed corn (finely ground or steam-flaked) enhances starch digestibility, thereby maximizing propionate production and glucose precursor availability, which supports gluconeogenesis and reduces the magnitude of negative energy balance [145]. This increased glucose availability not only supports lactose synthesis for milk production but also helps suppress excessive adipose tissue lipolysis by maintaining insulin secretion, thereby reducing NEFA mobilization and subsequent ketone body formation.

Critically, dietary management must rigorously avoid the inclusion of low-quality fermented feeds, particularly silages containing elevated concentrations of butyric acid. Butyrate represents a potent ketogenic precursor because it is efficiently converted to β-hydroxybutyrate during absorption across the rumen epithelium, thereby directly contributing to blood ketone body concentrations independent of hepatic ketogenesis [132]. Consequently, silages exhibiting poor fermentation quality—characterized by high butyrate content typically resulting from clostridial fermentation under anaerobic conditions with inadequate acidification—can dramatically increase ketosis incidence even when other nutritional parameters are optimized [132]. Therefore, forage quality assessment should routinely include fermentation acid profiles, and silages containing butyrate concentrations exceeding 0.5% of dry matter should be excluded from transition cow diets or used only in limited quantities with appropriate dilution [132].

#### 5.1.4. Dynamic Diet Adjustment and Individualized Nutrition

Recognizing that nutritional requirements vary substantially among individual cows based on parity, body condition, milk production genetics, and health status, feed formulations should not be implemented as static prescriptions applied uniformly to all animals. Rather, dietary management must incorporate dynamic adjustment protocols that respond to changing physiological conditions and individual variation [146]. For instance, primiparous heifers typically require different nutritional strategies compared to mature cows due to their continued growth, smaller rumen capacity, and different metabolic priorities [147]. Similarly, cows in different body condition categories may benefit from individualized nutritional plans that account for their differing energy reserves and mobilization patterns. Advanced dairy operations increasingly employ precision feeding technologies, including individual cow monitoring systems and group-specific diet delivery, to tailor nutritional interventions to specific subpopulations or even individual animals, thereby optimizing metabolic outcomes and minimizing disease risk.

Furthermore, dietary transitions should be implemented gradually and systematically rather than abruptly, as sudden dietary changes provoke rumen microbial disruption, reduce feed intake, and exacerbate metabolic stress [148]. Close-up diets should closely resemble fresh cow lactation diets in ingredient composition and nutrient density, differing primarily in inclusion rates rather than ingredient types, thereby minimizing the metabolic challenge associated with the peripartum dietary transition [149].

#### 5.1.5. Micronutrient Supplementation and Metabolic Support

Beyond macronutrient management, timely and adequate supplementation of essential trace minerals and vitamins plays a crucial role in maintaining metabolic health and preventing ketosis-associated metabolic disorders. Several micronutrients merit particular attention due to their involvement in energy metabolism, antioxidant defense, and metabolic regulation. Iodine, an essential component of thyroid hormones, influences basal metabolic rate and energy partitioning, with deficiency potentially impairing metabolic adaptation to lactation [150]. Phosphorus serves as a critical component of high-energy phosphate compounds (ATP, creatine phosphate) and participates in numerous metabolic pathways, including gluconeogenesis and fatty acid metabolism; phosphorus deficiency can impair energy metabolism and exacerbate negative energy balance [151]. Cobalt functions as the central atom in vitamin B12 (cobalamin), which serves as an essential cofactor for propionate metabolism—the primary gluconeogenic pathway in ruminants—and methylmalonic acid metabolism; cobalt deficiency impairs propionate utilization for gluconeogenesis and can precipitate or worsen ketosis [152]. Additionally, other trace minerals, including selenium, zinc, copper, and manganese, support antioxidant enzyme function, immune competence, and metabolic regulation, thereby indirectly influencing ketosis susceptibility through effects on oxidative stress management and overall metabolic health [153]. Consequently, transition cow diets must provide adequate but balanced supplementation of trace minerals, typically delivered through chelated or protected forms that enhance bioavailability and minimize antagonistic interactions [154].

#### 5.1.6. Glucogenic Precursor Supplementation and Metabolic Modifiers

Beyond conventional nutritional management, targeted supplementation with specific glucogenic precursors and metabolic modifiers during high-risk periods represents an evidence-based strategy for ketosis prevention that directly addresses the fundamental metabolic deficits underlying disease pathogenesis. Sodium propionate, administered as a dietary additive or oral drench, provides a direct source of propionate that bypasses ruminal fermentation and enters hepatic metabolism, where it serves as an immediate substrate for gluconeogenesis [155]. By increasing circulating glucose concentrations and enhancing glucose availability for lactose synthesis, propionate supplementation reduces the metabolic drive for excessive fat mobilization and ketogenesis, thereby directly interrupting the pathogenic cascade leading to ketosis. Clinical trials have consistently demonstrated that periparturient propionate supplementation—typically delivered as 250–500 g of sodium propionate daily during the final week prepartum and first two weeks postpartum—significantly reduces both subclinical and clinical ketosis incidence while improving energy balance and production outcomes [156].

Monensin supplementation, particularly during the transition period, is recognized as a highly effective nutritional intervention for ketosis prevention, operating primarily through its role as a ruminal modulator [157,158,159]. First, monensin acts as a carboxylic polyether ionophore that selectively inhibits the growth of Gram-positive bacteria within the rumen [160,161,162]. This selective pressure alters the microbial ecosystem, shifting the volatile fatty acid (VFA) profile towards enhanced propionate production. Second, this shift in fermentation markedly improves the energetic efficiency of the diet. The increased propionate yield simultaneously reduces carbon losses (methane and carbon dioxide excretion), thereby enhancing overall feed utilization efficiency [163]. Third, since propionate is the principal precursor for hepatic gluconeogenesis in ruminants, the increased supply of this VFA directly augments the cow’s glucose availability [164]. This enhanced glucose supply is critical for relieving the severity of the NEB that underlies ketosis [165]. Collectively, these metabolic adjustments position monensin as a powerful preventative tool, with administration via a controlled-release ruminal capsule (ensuring a stable dose for high-risk cows) or as a feed additive being common practice, typically at a safe prophylactic dose of 300–400 mg per cow per day [157,158,159].

Niacin (vitamin B3) supplementation represents another metabolic modifier with demonstrated efficacy in ketosis prevention, operating through multiple complementary mechanisms. First, niacin functions as an antilipolytic agent, directly suppressing hormone-sensitive lipase activity in adipocytes and thereby reducing NEFA mobilization from adipose tissue [166]. This antilipolytic effect reduces hepatic NEFA influx, mitigating both fatty liver development and excessive ketogenesis. Second, niacin enhances insulin sensitivity and glucose utilization in peripheral tissues, improving whole-body energy metabolism [167]. Third, niacin may exert direct hepatoprotective effects and influence hepatic lipid metabolism [168]. Collectively, these mechanisms position niacin as a valuable preventive supplement, with research indicating that supplementation at 12–24 g per day during the periparturient period reduces ketosis incidence and improves metabolic profiles [168].

Rumen-protected lipids—dietary fat sources that resist ruminal biohydrogenation and deliver fatty acids for intestinal absorption—provide energy-dense supplementation that can ameliorate negative energy balance without provoking rumen dysfunction or metabolic acidosis. By supplying concentrated energy in a form that does not require ruminal fermentation, protected lipids increase total energy intake and improve energy balance, thereby reducing the metabolic drive for fat mobilization and ketogenesis. However, lipid supplementation must be implemented judiciously, as excessive fat intake (>6–7% of diet dry matter) can suppress dry matter intake and impair fiber digestibility, potentially negating the intended benefits [169]. Optimal lipid supplementation strategies typically employ rumen-inert fat sources (calcium salts of long-chain fatty acids, hydrogenated triglycerides) at inclusion rates of 2–4% of diet dry matter, integrated into well-balanced transition diets. A comprehensive summary of integrated preventive approaches to bovine ketosis management is provided in Table 2.

Effective prevention strategies for bovine ketosis must be precisely differentiated, reflecting the distinct underlying pathophysiology of Type I and Type II diseases. Type I ketosis, which typically occurs several weeks postpartum, can be effectively mitigated through nutritional interventions aimed at maximizing dry matter intake and improving energy density during early lactation [24]. Specifically, these interventions involve optimizing transition diets, ensuring adequate provision of glucogenic precursors, and providing timely supplementation with propylene glycol to enhance hepatic gluconeogenesis and mitigate excessive ketone body production [140,166]. In contrast, Type II ketosis usually develops during the periparturient period and is closely associated with excessive body condition (BCS) at calving [25]. Preventive strategies for Type II ketosis, therefore, prioritize controlling BCS during late lactation and the dry period to prevent overconditioning, thus promoting metabolic adaptation through controlled energy restriction prepartum [97,131,133]. In addition, management practices that support liver function and reduce lipid mobilization, such as balanced prepartum nutrition and careful monitoring of high-risk cows, are essential for minimizing the incidence of Type II ketosis [140,144].

## 6. Therapeutic Interventions for Clinical Ketosis Management

Despite optimal implementation of preventive strategies, some dairy cows inevitably develop clinical ketosis due to individual metabolic susceptibility, management challenges, or unforeseen stressors that precipitate severe negative energy balance. When clinical ketosis occurs, prompt recognition and aggressive therapeutic intervention are essential to restore metabolic homeostasis, prevent progression to more severe metabolic decompensation, and minimize the adverse impacts on animal welfare, productivity, and longevity. Therapeutic approaches for ketosis management employ multiple complementary strategies targeting different aspects of the metabolic dysfunction, including acute restoration of blood glucose concentrations, modulation of hormonal signals regulating lipolysis and gluconeogenesis, management of neurological manifestations in severe cases, and supportive care addressing secondary complications. The following subsections present evidence-based therapeutic protocols organized according to their primary mechanisms of action and clinical applications.

### 6.1. Glucose Administration and Immediate Metabolic Stabilization

The fundamental metabolic derangement in clinical ketosis is severe hypoglycemia resulting from inadequate gluconeogenesis relative to glucose utilization for lactose synthesis and other essential metabolic processes [170]. Blood glucose concentrations in severely ketotic cows frequently decline to 30–40 mg/dL or below—well beneath the normal physiological range of 45–75 mg/dL—creating an immediate metabolic emergency that demands rapid intervention [171]. Consequently, the most direct therapeutic intervention involves intravenous administration of hypertonic glucose solutions (25–50% dextrose) at doses of 250–500 g per treatment, typically delivered slowly via jugular venipuncture to rapidly restore blood glucose concentrations. However, the therapeutic effect is characteristically transient, with blood glucose returning to hypoglycemic levels within 6–12 h due to continued glucose demands for milk synthesis and inadequate endogenous gluconeogenesis. Therefore, repeated glucose therapy over several days is often necessary until clinical improvement is observed [172].

Importantly, rapid intravenous glucose administration carries inherent metabolic risks that demand clinical awareness and management. Specifically, acute hyperglycemia stimulates substantial insulin secretion from pancreatic β-cells, and this insulin surge can trigger several adverse consequences [173]. First, elevated insulin concentrations promote cellular glucose uptake and glycogen synthesis, potentially leading to rebound hypoglycemia when the insulin effect peaks after glucose infusion is complete [174]. Second, rapid shifts in blood glucose and insulin concentrations can disrupt electrolyte homeostasis, particularly causing acute hypokalemia as insulin-stimulated cellular glucose uptake is accompanied by potassium influx into cells [175]. Third, abrupt osmotic shifts associated with hypertonic glucose infusion can provoke fluid redistribution and electrolyte imbalances [176]. Therefore, when employing intravenous glucose therapy, concurrent electrolyte supplementation is essential to prevent iatrogenic complications. Comprehensive metabolic support should include administration of balanced electrolyte solutions containing potassium, sodium, chloride, and ideally calcium and magnesium, either admixed with glucose solutions or administered as separate infusions, thereby maintaining electrolyte balance and preventing secondary metabolic derangements.

Beyond immediate glucose replacement, nutritional supplementation with B-complex vitamins and folic acid provides metabolic cofactors that facilitate energy metabolism and support recovery from ketosis. B vitamins, particularly thiamine (B1), riboflavin (B2), niacin (B3), pantothenic acid (B5), pyridoxine (B6), and cobalamin (B12), serve as essential cofactors for numerous enzymes involved in carbohydrate metabolism, fatty acid oxidation, and energy production [177,178]. Thiamine functions as a cofactor for pyruvate dehydrogenase and α-ketoglutarate dehydrogenase, critical enzymes in glucose oxidation and citric acid cycle function [179]. Riboflavin and niacin participate in the electron transport chain function as components of FAD and NAD, respectively, thereby supporting ATP production [180]. Pantothenic acid serves as a component of coenzyme A, essential for fatty acid metabolism and citric acid cycle function [181]. Cobalamin functions as a cofactor for methylmalonyl-CoA mutase, the enzyme converting methylmalonyl-CoA (derived from propionate) to succinyl-CoA for entry into the citric acid cycle, thereby directly supporting gluconeogenesis from propionate—the primary glucogenic pathway in ruminants [182]. Folic acid participates in one-carbon metabolism and nucleotide synthesis, supporting cellular proliferation and metabolic function [183]. Consequently, parenteral or oral supplementation with B-complex vitamins and folic acid can enhance energy metabolism, promote recovery of blood glucose homeostasis, and support overall metabolic recovery. These vitamins are typically administered via intramuscular injection or oral drench at therapeutic dosages (typically 2–5 times maintenance requirements) for 3–5 consecutive days during acute ketosis treatment.

Oral administration of readily absorbable carbohydrates complements parenteral glucose therapy by providing sustained glucose substrate availability through intestinal absorption. Oral drenching with glucose powder, dextrose monohydrate, or propylene glycol (a glucogenic alcohol readily converted to glucose in the liver) at dosages of 250–500 g per day provides additional glucose precursors that supplement endogenous gluconeogenesis and reduce dependence on repeated intravenous infusions [164]. Propylene glycol is particularly favored for oral glucose precursor supplementation due to its palatability, efficient absorption, and effective conversion to glucose in hepatic metabolism, with clinical trials demonstrating that daily oral propylene glycol administration at 300–500 g per day for 5–7 days significantly accelerates recovery from clinical ketosis [164]. Similarly, oral administration of cornstarch or other digestible carbohydrates provides glucose substrate for ruminal fermentation and subsequent propionate production, thereby supporting endogenous gluconeogenesis through physiological pathways [184].

### 6.2. Hormonal and Metabolic Modulation Therapies

Beyond direct glucose supplementation, therapeutic manipulation of hormonal signals regulating glucose homeostasis and lipid metabolism can provide additional metabolic benefits in ketosis management, particularly in cases exhibiting severe metabolic derangement or inadequate response to glucose therapy alone.

Insulin administration represents a paradoxical but physiologically rational therapeutic approach for ketosis management. Although ketotic cows characteristically exhibit relative insulin deficiency or insulin resistance contributing to excessive lipolysis and impaired glucose utilization, exogenous insulin therapy—when carefully administered alongside glucose supplementation—can enhance peripheral tissue glucose uptake, promote glycogen synthesis, suppress excessive lipolysis, and improve overall metabolic efficiency. The therapeutic rationale derives from insulin’s multiple beneficial metabolic effects: it promotes cellular glucose utilization for energy production; stimulates glycogen storage, providing a glucose reserve; suppresses hormone-sensitive lipase activity, thereby reducing NEFA mobilization; and enhances protein synthesis, supporting recovery [178]. However, insulin therapy must be implemented with extreme caution due to the substantial risk of severe hypoglycemia if insulin is administered without adequate concurrent glucose provision. Therefore, insulin is typically administered only in conjunction with intravenous glucose infusion, using low doses (0.1–0.2 IU per kg body weight) of regular insulin given subcutaneously or intramuscularly immediately following glucose infusion, with close monitoring of blood glucose concentrations at 2–4-h intervals to detect and manage potential hypoglycemia.

Corticosteroid therapy, employing synthetic glucocorticoids such as dexamethasone or prednisolone, exerts multiple metabolic effects that can benefit ketotic cows through several complementary mechanisms [185]. First, glucocorticoids potently suppress adipose tissue lipolysis by inhibiting hormone-sensitive lipase and reducing β-adrenergic receptor sensitivity, thereby reducing NEFA mobilization and decreasing hepatic lipid influx [186]. Second, corticosteroids reduce hepatic ketone body synthesis by modulating the expression and activity of ketogenic enzymes. Third, glucocorticoids stimulate hepatic glycogenolysis, liberating glucose from hepatic glycogen stores and acutely elevating blood glucose concentrations [187]. Fourth, corticosteroids enhance gluconeogenesis by promoting the expression of key gluconeogenic enzymes (PEPCK, G6PC) and providing amino acid substrates through protein catabolism [188]. Collectively, these effects—reduced lipolysis, decreased ketogenesis, and increased glucose availability—can substantially improve metabolic status in ketotic cows.

However, corticosteroid therapy carries significant risks and contraindications that demand judicious use and careful patient selection. Prolonged or high-dose glucocorticoid administration causes profound immunosuppression by inhibiting lymphocyte proliferation, suppressing cytokine production, and impairing neutrophil function, thereby increasing susceptibility to infectious diseases, including mastitis, metritis, and pneumonia. Additionally, chronic corticosteroid exposure can induce insulin resistance, impair wound healing, promote muscle catabolism, and potentially precipitate other metabolic disorders [189]. Furthermore, corticosteroid administration during pregnancy—particularly during late gestation—can trigger premature parturition, posing risks to both cow and calf. Therefore, corticosteroid therapy for ketosis should be reserved for severe cases demonstrating inadequate response to conventional glucose and nutritional therapies, employed only for short durations (typically 1–3 doses over 24–72 h), and avoided in cows with concurrent infectious diseases or late pregnancy. Throughout corticosteroid treatment, close monitoring of the animal’s clinical condition, appetite, milk production, and response to therapy is essential, with strict control of drug dosage and treatment duration to minimize adverse effects while maximizing therapeutic benefits.

### 6.3. Management of Neurogenic Ketosis and Central Nervous System Manifestations

In a subset of clinical ketosis cases—estimated at 5–10% of affected animals—cows develop neurogenic or nervous ketosis, characterized by prominent central nervous system manifestations resulting from the effects of ketone bodies and metabolic acidosis on neural function [190]. These animals exhibit distinctive behavioral and neurological signs, including nervous excitement, hyperesthesia, muscle tremors, excessive salivation, apparent blindness, aggressive behavior, compulsive walking or circling, head pressing, and, in severe cases, seizures or coma [191]. Neurogenic ketosis represents a medical emergency requiring prompt recognition and specific therapeutic interventions to prevent self-injury, permanent neurological damage, or death.

The pathophysiology of neurogenic ketosis involves multiple mechanisms by which severe ketosis disrupts central nervous system function. Elevated ketone body concentrations, particularly acetone and acetoacetate, exert direct neurotoxic effects by disrupting neuronal membrane function and neurotransmitter metabolism [192]. Additionally, metabolic acidosis—resulting from accumulation of acidic ketone bodies—alters cerebrospinal fluid pH and ion gradients, disturbing neuronal excitability and synaptic transmission [193]. Severe hypoglycemia, invariably present in ketotic animals, deprives neurons of their primary energy substrate, impairing neuronal metabolism and potentially causing acute neuronal dysfunction [194]. Furthermore, electrolyte disturbances commonly accompanying ketosis, particularly hypocalcemia and hypomagnesemia, can independently contribute to neurological signs by affecting neuronal membrane potential and neuromuscular function [195].

Therapeutic management of neurogenic ketosis requires a multifaceted approach addressing both the underlying metabolic derangements and the acute neurological manifestations. In addition to the standard metabolic interventions described above (glucose administration, electrolyte supplementation, B-vitamin therapy), animals exhibiting pronounced nervous signs benefit from specific psychotropic medications that alleviate agitation, reduce neurological hyperexcitability, and promote behavioral normalization. Sedative or anxiolytic medications can be administered to reduce nervous excitement, prevent self-injury from compulsive behavior, and facilitate safe handling and treatment of the affected animal [196].

Among the available sedative options, chloral hydrate has historically demonstrated particular efficacy in managing neurogenic ketosis and merits specific discussion. Chloral hydrate functions as a central nervous system depressant, producing sedation, anxiolysis, and muscle relaxation through enhancement of GABAergic inhibitory neurotransmission [197]. Importantly, beyond its sedative properties, chloral hydrate exerts additional metabolic effects that uniquely benefit ketotic animals: it has been shown to stimulate ruminal production of propionic acid, the primary glucogenic volatile fatty acid in ruminants. By enhancing propionate production and absorption, chloral hydrate indirectly supports hepatic gluconeogenesis and glucose availability, complementing the direct glucose-raising effects of parenteral glucose therapy [198]. This dual mechanism—central nervous system sedation combined with enhanced glucogenic precursor production—positions chloral hydrate as an ideal therapeutic agent for neurogenic ketosis. Chloral hydrate is typically administered as an oral drench or stomach tube at dosages of 60–120 g per cow, diluted in water to prevent gastric irritation, with sedative effects manifesting within 30–60 min and persisting for 4–8 h. Treatment can be repeated after 12–24 h if neurological signs persist, although most cases show substantial improvement after one or two treatments when combined with comprehensive metabolic support.

Alternative sedative options for managing neurogenic ketosis include xylazine (an α2-adrenergic agonist providing sedation and analgesia), acepromazine (a phenothiazine tranquilizer), or diazepam (a benzodiazepine anxiolytic), each selected based on the severity of neurological signs, concurrent health conditions, and clinical judgment [199]. Regardless of the specific sedative employed, the primary therapeutic goals remain consistent: to ensure animal safety, facilitate medical treatment, and allow time for metabolic recovery to resolve the underlying neurological dysfunction.

### 6.4. Supportive Care and Management of Complications

Throughout the treatment process, comprehensive supportive care and nutritional management are equally critical to therapeutic pharmacological interventions in determining recovery outcomes. Maintaining balanced nutrition for the ketotic cow requires careful attention to feed palatability, feeding frequency, and nutrient composition [200]. Affected cows typically exhibit severe anorexia or inappetence as a primary clinical sign, and restoring voluntary feed intake represents a crucial therapeutic objective. Strategies to encourage feed consumption include offering highly palatable, energy-dense feeds in small, frequent portions; providing fresh feed multiple times daily to stimulate appetite; ensuring constant access to clean, fresh water; and considering oral appetite stimulants or flavor enhancers when voluntary intake remains inadequate [201]. The diet provided to recovering ketotic cows should be carefully formulated to ensure adequate but not excessive intake of protein, vitamins, and minerals while providing sufficient energy density to support metabolic recovery without overwhelming digestive capacity [202]. Total mixed rations designed for fresh cows, potentially supplemented with additional palatable ingredients such as molasses, wet brewers’ grains, or fresh green forage, can stimulate appetite and support recovery.

Equally important, prompt recognition and aggressive treatment of potential secondary complications and concurrent diseases are vital to overall case management and recovery success. Ketosis rarely occurs in isolation; rather, it frequently develops alongside or precipitates other periparturient disorders that demand concurrent therapeutic attention [203]. Hepatic lipidosis (fatty liver disease) represents an almost universal finding in clinical ketosis cases, resulting from the same metabolic processes—excessive NEFA mobilization and hepatic lipid accumulation—that drive ketogenesis [204]. While fatty liver is managed primarily through resolution of the underlying negative energy balance and ketosis, severe cases may benefit from specific hepatoprotective interventions, including choline supplementation (which supports hepatic VLDL synthesis and lipid export), antioxidant therapy (vitamin E and selenium to reduce oxidative damage), and possibly lipotropic agents.

Displaced abomasum, particularly left displaced abomasum (LDA), exhibits strong epidemiological and pathophysiological associations with ketosis, likely due to hypomotility and abomasal gas accumulation resulting from metabolic acidosis and altered gastrointestinal function [205]. When abomasal displacement is diagnosed concurrent with ketosis, surgical correction of the displacement must be prioritized alongside medical ketosis management, as a displaced abomasum perpetuates anorexia and prevents restoration of normal energy balance [206].

Metritis and retained placenta frequently accompany or predispose to ketosis due to shared risk factors, including immunosuppression, metabolic stress, and inflammatory responses that impair feed intake and exacerbate negative energy balance [207]. Active uterine infections demand prompt antibiotic therapy, anti-inflammatory medication, and supportive care to resolve infection and prevent progression to systemic disease.

Mastitis incidence increases substantially in ketotic cows due to immunosuppression associated with negative energy balance, elevated cortisol, and NEFA-induced neutrophil dysfunction [208]. Clinical mastitis cases require appropriate antimicrobial therapy, frequent udder examination and milk culture, and aggressive supportive care including anti-inflammatory medication and fluid therapy [209].

Hypocalcemia (milk fever) and ketosis frequently coexist, as both disorders commonly occur during the periparturient period and share certain risk factors [210]. When clinical signs suggest concurrent hypocalcemia (weakness, recumbency, decreased rumen motility), immediate calcium therapy via intravenous or subcutaneous administration of calcium solutions is essential, as hypocalcemia both exacerbates ketosis through reduced feed intake and represents a life-threatening emergency in its own right [17,211,212]. Specifically, the lack of Ca^+^ significantly reduces smooth muscle contraction, leading to marked rumen stasis and decreased intestinal motility [17,180,213]. This reduction in dry matter intake (DMI) directly restricts energy availability, thereby intensifying the necessary reliance on adipose mobilization and subsequent ketogenesis [211]. Furthermore, severe hypocalcemia represents a life-threatening emergency in its own right. Consequently, timely calcium supplementation is critically required to address the dual threat of acute clinical hypocalcemia and the profound metabolic destabilization it imposes on pre-existing ketosis [213].

Therapeutic approaches for bovine ketosis should be precisely tailored to the distinct metabolic characteristics of Type I and Type II ketosis. Type I ketosis, which generally occurs several weeks after calving, is typically responsive to treatments that directly increase circulating glucose availability and suppress excessive ketogenesis [24]. Standard therapeutic interventions include oral administration of glucogenic precursors such as propylene glycol, intravenous glucose infusions in more severe cases, and short-term use of glucocorticoids to stimulate hepatic gluconeogenesis and reduce milk yield-associated glucose demand [164,170,172]. Auxiliary supplementation with B vitamins (Vitamin B12 for propionate metabolism) is often included to enhance energy utilization [178,179,180,181,182]. In contrast, Type II ketosis develops predominantly during the periparturient period and requires a treatment focus on correcting underlying metabolic dysregulation rather than solely increasing glucose supply. In addition to glucogenic supplementation, supportive therapies aimed at improving hepatic function, such as supplementation with lipotropic agents (rumen-protected choline and methionine) to facilitate VLDL export, limiting further fat mobilization, and enhancing insulin sensitivity are critical [175,176,187,204]. Early identification of high-risk cows and prompt intervention are essential to prevent progression to severe fatty liver and to improve therapeutic outcomes.

Based on the above findings, it can be concluded that effective therapeutic management of clinical ketosis requires integrated implementation of multiple interventions addressing acute glucose deficiency, hormonal dysregulation, neurological manifestations, nutritional support, and concurrent diseases. This comprehensive approach, combining parenteral glucose therapy, metabolic modulation, symptomatic treatment, nutritional management, and vigilant monitoring for complications, maximizes recovery rates, minimizes case duration, and optimizes long-term productive outcomes for affected animals. Therapeutic interventions for clinical ketosis management are detailed in Table 3.

## 7. Conclusions and Future Directions

Ketosis remains a critical metabolic disorder threatening dairy cow health and farm profitability during the periparturient period. This review has highlighted that negative energy balance and hepatic NEFA overload constitute the fundamental pathophysiological drivers, while genetic variation in key metabolic genes influences individual susceptibility. Although current prevention strategies emphasizing nutritional management show promise, precise control remains elusive. Future research should prioritize the integration of multi-omics approaches to map complete regulatory networks linking metabolic, hormonal, and inflammatory pathways. Validation of candidate gene functions through targeted knockout and overexpression studies is essential. Development of practical early-stage biomarkers, potentially via milk metabolomics or wearable sensors, would facilitate real-time monitoring and timely intervention. Genomic selection programs incorporating ketosis resistance should be expanded, while precision nutrition strategies tailored to individual genetic profiles warrant investigation. Ultimately, translating molecular discoveries into integrated management frameworks combining genetic improvement, predictive diagnostics, and targeted interventions will revolutionize periparturient health management and enhance both animal welfare and economic sustainability in dairy production.

## Figures and Tables

**Table 1:** Functional classification of key genes and signaling pathways associated with ketosis in dairy cows.

Functional Category	Gene/Pathway	Key Role in Ketosis	Tissue/Site	References
Lipid Mobilization and Fatty Acid Oxidation	*ACSL1*	Activates long-chain fatty acids to acyl-CoA, providing substrate for β-oxidation; upregulated in ketotic cattle	Liver, Adipose tissue	[56,57,58,59,60]
	*CPT1A*	Rate-limiting enzyme for transport of long-chain fatty acids into mitochondria for β-oxidation; upregulated	Liver	[56,57,58,74]
	*CPT2*	Works with CPT1A in mitochondrial fatty acid import for oxidation; upregulated	Liver	[56,57,58]
	*ACOX1*	Peroxisomal fatty acid oxidase involved in very-long-chain fatty acid oxidation	Liver	[58]
	*ACAA2*	Mitochondrial thiolase participating in final steps of β-oxidation	Liver	[58]
	*FABP1*	Liver fatty acid binding protein; facilitates intracellular fatty acid transport and activation; upregulated in ketotic cows	Liver	[58]
	*CD36*	Fatty acid transporter mediating NEFA uptake; overexpression exacerbates hepatic lipid accumulation and lipotoxicity	Hepatocytes	[110]
Ketone Body Synthesis and Utilization	*HMGCS2*	Rate-limiting enzyme of ketogenesis; determines BHBA production capacity; upregulated	Liver	[67,68,89,90,91,111,112]
	*ACAT1*	Catalyzes condensation of two acetyl-CoA to acetoacetyl-CoA, providing precursor for ketogenesis	Liver	[67,111,112]
	*HMGCL*	Cleaves HMG-CoA to acetoacetic acid (AcAc), precursor for BHBA and acetone	Liver	[69]
	*BDH1*	Mediates reversible conversion between AcAc and BHBA, regulating ketone body ratio	Liver	[113]
	*OXCT1*	Key enzyme for ketone utilization in extrahepatic tissues; reduced expression may limit ketone clearance	Extrahepatic tissues (muscle)	[70]
Lipid Synthesis and Triglyceride Accumulation	*SREBP1/SREBP1c*	Transcription factors that promote lipogenic gene expression (ACC1, FAS, SCD1); upregulated in ketosis	Liver	[58,71]
	*ACC1 (ACACA)*	Catalyzes malonyl-CoA formation; key step in fatty acid synthesis; upregulated	Liver	[58,75]
	*FAS (FASN)*	Fatty acid synthase; catalyzes palmitate synthesis during lipogenesis	Liver	[58]
	*SCD1*	Stearoyl-CoA desaturase; introduces double bonds in fatty acids; linked to lipid composition changes	Liver	[58]
	*DGAT1*	Diacylglycerol acyltransferase 1; final enzyme in TG synthesis; associated with ketosis susceptibility	Liver	[33,65]
	*DGAT2*	Diacylglycerol acyltransferase 2; contributes to TG synthesis and hepatic lipid storage	Liver	[58]
Key Metabolic and Regulatory Signaling Pathways	*PPARα*	Nuclear receptor promoting fatty acid oxidation and ketogenesis; upregulated in ketosis	Liver	[74,89,90,91]
	*INSR*	Insulin receptor; modulates insulin sensitivity and downstream AKT signaling, influencing lipolysis	Liver, Adipose tissue	[74]
	*IRS1*	Insulin receptor substrate 1; mediates insulin signaling to AKT, affects glucose and lipid metabolism	Adipose tissue, Skeletal muscle, Liver	[74]
	*AKT1*	Serine-threonine kinase in insulin signaling pathway; modulates glucose uptake and lipid metabolism	Adipose tissue, Skeletal muscle, Liver	[74]
	*MAPK1*	MAP kinase; BHB induces MAPK1 upregulation leading to hepatic lipotoxicity and lipid metabolism disorder	Liver	[99,114]
	*FGF21*	Endocrine regulator of metabolism; imbalance with MAPK1 implicated in hepatic lipid disorders	Liver, Circulation	[99,114]
	*TFEB*	Transcription factor regulating autophagy/lysosome; increased activity promotes adipocyte lipolysis	Adipose tissue	[83]
	*NDUFAB1*	Mitochondrial metabolic regulator; activation mitigates NEFA-induced cytotoxicity in adipocytes	Adipocytes	[84]
Oxidative Stress and Apoptosis Response	*SOD2*	Mitochondrial superoxide dismutase; upregulated in response to elevated BHB (0.8–1.2 mmol/L)	Liver	[66]
	*HIF-2α (EPAS1)*	Stabilized by cellular stress; promotes lipid uptake and accumulation via ATF4 activation	Hepatocytes	[115]
	*ATF4*	ER stress-related transcription factor activated downstream of PERK; promotes lipid uptake/accumulation	Hepatocytes	[105]
	*p53 (TP53)*	Pro-apoptotic transcription factor increased by ROS–p38–p53 axis leading to hepatocyte apoptosis	Liver	[95,96,97,98]
	*Nrf2 (NFE2L2)*	Antioxidant response regulator; decreased expression observed with high BHB leading to reduced cytoprotection	Liver	[96,97,98]
	*SIRT3*	Mitochondrial deacetylase that activates AMPK pathway and mitigates oxidative stress-induced apoptosis	Mammary epithelial cells, Liver	[105]
	*AMPK*, *mTOR*, *FoxO*, *PERK–eIF2α*, *ROS–p38–p53/Nrf2*	Core signaling pathways integrating energy sensing, autophagy, ER stress and oxidative responses in ketosis	Adipose tissue, Skeletal muscle, Liver, Mammary gland	[85,86,87,88,89,90,91,92,93,94,95,96,97,98,99,105,114]

**Table 2:** Integrated preventive strategies for bovine ketosis management.

Strategy Category	Specific Intervention	Key Mechanisms/Benefits	Implementation Details	References
Nutritional Management—Prepartum	Body Condition Score Control	Prevents excessive fat mobilization and lipolysis; reduces NEB severity	Maintain BCS 3.0–3.5 at calving; control energy intake during the far-off dry period (60–21 days before calving)	[131,133]
	Close-up Diet Transition	Prepares rumen microbiome for lactation diet; enhances metabolic adaptation	Gradually increase concentration from 30–35% to 40–50% during the final 21 days prepartum	[97]
Environmental Optimization	Stocking Density Management	Reduces stress and competition; improves feed intake	Maintain < 80% pen capacity in transition facilities; provide min. 30 inches bunk space/cow	[89,135]
	Thermal Stress Control	Prevents suppression of feed intake; reduces maintenance energy requirements	Provide shade, fans, evaporative cooling (heat); adequate bedding and wind protection (cold)	[136,138]
Dietary Composition	Forage-to-Concentrate Balance	Optimizes energy density while maintaining rumen health	Formulate diets with 40–55% concentrate (DM basis); adjust based on forage quality	[140]
	Protein Management	Avoids the metabolic burden of excess protein catabolism; maintains adequate supply	Control crude protein to 14–16% of diet DM; avoid excess > 17–18%	[141,142,143]
	Glucogenic Precursor Supply	Provides substrate for gluconeogenesis; reduces ketogenic substrate excess	Include adequate starch sources; ensure propionate production from fermentation	[144,145]
	Silage Quality Control	Prevents butyrate-induced ketogenesis	Exclude silages with butyrate > 0.5% DM; ensure proper fermentation quality	[132]
Micronutrient Supplementation	Trace Minerals	Support energy metabolism, antioxidant defense, immune function	Adequate iodine (thyroid function), phosphorus (ATP synthesis), cobalt (B12/propionate metabolism), Se, Zn, Cu, Mn	[150,151,152,153,154]
	Vitamins	Essential cofactors for metabolic pathways	Ensure adequate vitamin A, D, E supplementation; consider B-vitamin fortification	[166]
Metabolic Modifiers	Sodium Propionate	Direct gluconeogenic substrate; increases blood glucose; reduces fat mobilization	250–500 g/day during the final week prepartum and the first 2 weeks postpartum	[155,156]
	Monensin	Increases gluconeogenic precursor; Augments glucose availability; Prevents ketogenesis	300–400 mg/day	[157,158,159,160,161,162,163,165]
	Niacin Supplementation	Antilipolytic effect; suppresses NEFA mobilization; enhances insulin sensitivity	12–24 g/day during the periparturient period	[166,167,168]
	Rumen-Protected Lipids	Energy-dense supplementation; improves energy balance without rumen dysfunction	Include at 2–4% of diet DM; use calcium salts or hydrogenated triglycerides	[169]
Dynamic Management	Individualized Nutrition	Accounts for variation in requirements based on parity, body condition, genetics	Separate primiparous from multiparous; adjust diets by BCS category; use precision feeding	[146,147]
	Gradual Diet Transitions	Prevents rumen microbial disruption; maintains feed intake	Implement dietary changes progressively; close-up diets should resemble fresh cow diets	[148,149]

**Table 3:** Therapeutic interventions for the management of clinical ketosis in dairy cows.

Therapeutic Category	Agent/Intervention	Mechanism of Action	Dosage and Administration	References
Immediate Glucose Restoration	Intravenous Dextrose	Direct blood glucose elevation; immediate metabolic stabilization	250–500 g per treatment as a 25–50% solution via slow IV; repeat as needed for several days	[170,171,172]
	Electrolyte Supplementation	Prevents iatrogenic complications; maintains electrolyte balance during glucose therapy	Concurrent administration of K, Na, Cl, Ca, Mg solutions with glucose therapy	[175,176]
Oral Glucose Precursors	Propylene Glycol	Glucogenic alcohol converted to glucose in liver; sustained substrate availability	300–500 g/day oral drench for 5–7 days	[164]
	Glucose/Dextrose Powder	Direct carbohydrate substrate; supplements endogenous gluconeogenesis	250–500 g/day oral administration	[164]
	Cornstarch/Digestible CHO	Ruminal fermentation to propionate; physiological gluconeogenesis support	Include in oral drench or feed; variable dosing	[184]
Vitamin Supplementation	B-Complex Vitamins	Essential cofactors for energy metabolism (thiamine, riboflavin, niacin, B12, etc.)	IM injection or oral drench at 2–5× maintenance for 3–5 days	[178,179,180,181,182]
	Folic Acid	Supports one-carbon metabolism and cellular function	Therapeutic dosage IM or oral for 3–5 days	[183]
Hormonal Modulation	Insulin + Glucose	Enhances glucose utilization; suppresses lipolysis; promotes glycogen storage	CAUTION: Only with adequate concurrent glucose; requires careful monitoring	[178]
	Glucocorticoids	Stimulates gluconeogenesis; anti-inflammatory; appetite stimulation	Variable protocols; single IM injection (dexamethasone); monitor for adverse effects	[185,186,187,188,189]
Neurogenic Ketosis Management	Chloral Hydrate	CNS sedation; enhances ruminal propionate production (dual mechanism)	60–120 g oral drench diluted in water; repeat after 12–24 h if needed	[197,198]
	Alternative Sedatives	Xylazine: sedation/analgesia; Acepromazine: tranquilization; Diazepam: anxiolysis	Selected based on severity and clinical judgment; standard veterinary dosing	[196,199]
Supportive Care	Nutritional Support	Restores voluntary feed intake; provides energy for recovery	Offer highly palatable, energy-dense feeds in small frequent portions; ensure fresh water	[200,201,202]
	Hepatoprotective Therapy	Supports hepatic VLDL synthesis; reduces oxidative damage	Choline supplementation; vitamin E and selenium; lipotropic agents	[204]
Complication Management	Hepatic Lipidosis	Resolution through underlying NEB and ketosis treatment	Addressed primarily through metabolic stabilization; specific hepatoprotection as above	[204]
	Displaced Abomasum	Surgical correction essential for recovery	Prioritize alongside medical ketosis management	[205,206]
	Metritis/Retained Placenta	Antibiotic and anti-inflammatory therapy	Prompt treatment of uterine infections; standard antibiotic protocols	[207]
	Mastitis	Antimicrobial therapy; supportive care	Culture-guided antibiotic selection; anti-inflammatory medication; fluid therapy	[208,209]
	Hypocalcemia	Immediate calcium replacement	IV or SC calcium solutions; treat as life-threatening emergency	[210,212]

## Data Availability

No new data were created or generated for this review article.
